# Routine contrast-enhanced CT is insufficient for TNM-staging of duodenal adenocarcinoma

**DOI:** 10.1007/s00261-022-03589-z

**Published:** 2022-07-21

**Authors:** G. Litjens, C. J. H. M. van Laarhoven, M. Prokop, E. J. M. van Geenen, J. J. Hermans

**Affiliations:** 1 Department of Medical Imaging, Radboud University Medical Center, Radboud Institute for Health Sciences, Nijmegen, The Netherlands; 2 Department of Surgery, Radboud University Medical Center, Radboud Institute for Health Sciences, Nijmegen, The Netherlands; 3 Department of Gastroenterology and Hepatology, Radboud University Medical Center, Radboud Institute for Health Sciences, Nijmegen, The Netherlands

**Keywords:** Duodenum, Adenocarcinoma, Neoplasm staging, Computed tomography, Diagnostic imaging

## Abstract

**Purpose:**

Adequate TNM-staging is important to determine prognosis and treatment planning of duodenal adenocarcinoma. Although current guidelines advise contrast-enhanced CT (CECT) for staging of duodenal adenocarcinoma, literature about diagnostic tests is sparse.

**Methods:**

In this retrospective single-center cohort study, we analyzed the real life performance of routine CECT for TNM-staging and the assessment of resectability of duodenal adenocarcinoma. Intraoperative findings and pathological staging served as reference standard for resectability, T-, and N-staging. Biopsies, ^18^FDG-PET-CT, and follow-up were used as the reference standard for M-staging.

**Results:**

Fifty-two consecutive patients with duodenal adenocarcinoma were included, 26 patients underwent resection. Half of the tumors were isodense to normal duodenum on CECT. The tumor was initially missed in 7/52 patients (13%) on CECT. The correct T-stage was assigned with CECT in 14/26 patients (54%), N-stage in 11/26 (42%), and the M-stage in 42/52 (81%). T-stage was underestimated in (27%). The sensitivity for detecting lymph node metastases was only 24%, specificity was 78%. Seventeen percent of patients had indeterminate liver or lung lesions on CECT. Surgery with curative intent was started in 32 patients, but six patients (19%) could not be resected due to unexpected local invasion or metastases.

**Conclusion:**

Radiologists and clinicians have to be aware that routine CECT is insufficient for staging and determining resectability in patients with duodenal adenocarcinoma. CECT underestimates T-stage and N-stage, and M-stage is often unclear, resulting in futile surgery in 19% of patients. Alternative strategies are required to improve staging of duodenal adenocarcinoma. We propose to combine multiphase hypotonic duodenography CT with MRI.

## Introduction

Duodenal adenocarcinoma (DA) is a rare malignancy; it represents less than 1% of all gastrointestinal tract tumors [1–3]. However, the incidence is increasing: in the United States of America (USA) the incidence of small intestine tumors has more than doubled from 4,700/year to 11,110/year in the past 20 years. However, data for DA specifically are not available for the USA [4, 5]. For the Netherlands, these data are available: with a more than fourfold increase of the incidence of DA from 46 patients in 2000 to 192 patients in 2020 [1, 2].

While DA represent over 50% of small bowel adenocarcinoma, they differ from other small bowel tumors: the resection rate of 41–57% for DA is substantially lower than the 83–95% resection rate for jejunal and ileal cancer, and the median overall survival (OS) of 13–40 months for DA compares unfavorable to 19–63 months for jejunal and ileal cancer [2, 3, 6, 7]. Because DA is a rare disease, the majority of publications that are available discuss all small bowel tumors as one group or include DA in the analysis of periampullary tumors [8–12].

Surgical resection is the only potentially curative treatment for DA. Without resection, 5-year survival is only 1%, after resection this increases to 46% [13]. The presence of lymph node (LN) metastases in the resection specimen reduces 5-year survival to 21% compared to 65% in patients without LN metastases [13]. Approximately one-third (34–38%) of patients with DA have distant metastasis at the time of diagnosis [2, 6], generally leading to palliative treatment.

DA [14]. New treatment strategies may include neoadjuvant treatment, similar to esophageal and rectal cancer [13, 15, 16], or local treatment of oligometastatic disease, similar to strategies in colorectal cancer [17, 18]. Adequate staging to select patients who could benefit from these treatment strategies is essential.

Two guidelines are currently available for small bowel adenocarcinoma: the 2017 French intergroup clinical practice guidelines [19] and the 2020 NCCN guideline version 2.2020 [20]. Recommendations for the diagnostic work-up in the French guideline are short and based on expert opinion only; they advocate CECT for primary staging. The newer NCCN guidelines on small bowel adenocarcinoma provide more detailed guidelines for the diagnostic work-up of DA. CECT is recommended as primary staging modality. EUS, MRI, and ^18^FDG-PET-CT are recommended as problem-solving tools if CT is equivocal. Recommendations are mostly based on studies on small bowel carcinoma. While both guidelines advise CECT as the primary staging modality, none of the two provides data about the accuracy of CECT for DA. In this study, we analyze the performance of CECT in the diagnostic work-up for patients with DA at a tertiary referral hospital.

## Materials and methods

This is a retrospective single-center cohort study to determine the real life performance of routine CECT for TNM-staging and resectability assessment of duodenal adenocarcinoma. The institutional review board approved the study and waived informed consent.

### Patients

We identified all patients with DA at our institution between January 1st of 2000 and June 1st of 2020. To identify patients we searched the patient files using 11 synonyms for ‘duodenal neoplasm’ in Dutch and English. Patients with histopathologically proven DA and a CECT scan available were included. Patients were excluded if they had opted out for use of their data for research, if they had a tumor of the pancreas, bile duct, or ampulla of Vater, if the histologic subtype was not adenocarcinoma, if simultaneously another malignancy was present, or if time between CECT and surgery was over 8 weeks. Patients primarily diagnosed at our institution as well as referred patients were included.

### Data acquisition

Patient records were reviewed to obtain clinical data, including: age, sex, diagnostic modality the tumor was first detected with, CT parameters (contrast phases, use of oral contrast agent, slice thickness, scan year), and use of problem-solving tools (^18^FDG-PET-CT, MRI, and EUS). Patients who underwent surgery had the following variables collected: curative or palliative intent of surgery; if a resection was performed or not; and reason why resection was not performed (unexpected distant metastases or local invasion). Pathological TNM stage was retrieved from the pathology reports (TNM 8th edition, small Intestine: Adenocarcinoma, of the American Joint Committee on Cancer staging [21]).

### Imaging analysis

A radiologist with 20 years of experience in abdominal radiology retrospectively evaluated all CECT scans, blinded for the clinical data. All available contrast-phases were used for the evaluation of the CT scans. Evaluation included tumor location (duodenal segment), contrast-enhancement of the tumor in the portal-venous phase (PVP) compared to the healthy duodenal wall, and resectability of the tumor.

Resectability was classified as; resectable, borderline, and irresectable and was based on vessel contact with coeliac trunk, superior mesenteric artery (SMA), common hepatic artery (CHA), superior mesenteric vein (SMV), portal vein, inferior caval vein, and jejunal arteries and veins. Vessel contact was categorized as ≤ 90, 90–180, 180–270, and > 270 °. Criteria for resectability for pancreatic cancer of the Dutch Pancreatic Cancer Group (DPCG) [22] were used as a guideline, but because these criteria are not designed for DA and therefore suboptimal, final classification (resectable, borderline resectable, or irresectable) was according to expert opinion. The criteria from the DPCG are; resectable: no arterial contact and venous contact ≤ 90 °; borderline resectable: arterial contact ≤ 90 ° and/or venous contact 90°–270° and no occlusion; locally advanced indicated to be irresectable: arterial contact > 90° and/or venous contact > 270° or occlusion. Finally, clinical TNM stage (again 8th edition, small Intestine: Adenocarcinoma) was assessed on CECT. T1 and T2 were combined, because distinction between invasion of lamina propria, submucosa, and muscularis propria is not possible on CECT. Tumors that were not visible were classified as Tx. LNs with a short axis of ≥ 10 mm on axial orientation were considered malignant. We combined N1 and N2 to get a binary outcome that enables us to calculate sensitivity and specificity of CECT for the detection of LN metastases.

Only resected patients were included in the T- and N-stage analysis. The pathological T- and N-stage served as reference standard for comparison to the clinical T- and N-stage on CECT. All patients were included in the M-stage analysis, for which histopathological confirmation by biopsy, clinical confirmation with ^18^FDG-PET-CT, or growth during follow-up was used as reference standard. Indeterminate lesions on CECT (Mx), which could not be verified by the reference standard, remained Mx as final M-stage. After the radiologist analyzed all scans, a surgeon with 24 years of experience with pancreatic and hepato-biliary surgery evaluated resectability on CECT similarly as the radiologist (expert opinion, with the DPCG criteria as guideline). The surgeon was blinded for the given treatment, patient outcome and the radiologists’ assessment of resectability, but was informed about all other features evaluated by the radiologist.

### Statistical analysis

Data were analyzed using the IBM SPSS Statistics 25 software package (IBM Corporation). Continuous variables were summarized with standard descriptive statistics including mean, standard deviation, median, and range. Categorical variables were summarized with frequencies. A *p*-value below 0.05 was considered statistically significant. Accuracy of LN staging was calculated using cross-tabulations.

## Results

### Patient characteristics and diagnosis

Eighty-four patients with suspected DA were identified of which 52 patients met the inclusion criteria, 32 patients were excluded because the final diagnosis was not DA (*n* = 18), there was no CECT available (*n* = 4), the time between CECT and surgery was more than 8 weeks (*n* = 6), there was a simultaneous other malignancy (*n* = 3), or there was no histopathological prove (*n* = 1). None of the eligible patients had opted out for use of their data for research. Table 1 summarizes patient characteristics. In most patients endoscopy was the modality on which the diagnosis DA was first suspected, followed by CECT. In 13 patients, the diagnosis was initially missed at endoscopy (6/52, 12%), CECT (4/52, 8%), or both (3/52, 6%). The median delay between missed diagnosis and final diagnosis was 12 weeks (1–49 weeks) for endoscopy and five weeks (1–103 weeks) for CECT.

**Table 1:** Patient characteristics

Characteristic	
*Total number of patients*	52
Female, *n* (%)	28 (54%)
Male,*n* (%)	24 (46%)
Age, median in years (range)	69 (40–84)
*Tumor first detected on, n (%)*	
Endoscopy	32 (62%)
CECT	13 (25%)
Ultrasound	4 (8%)
^18^FDG-PET-CT	2 (4%)
MRI	1 (2%)
*Tumor location, n (%) (on CECT)*	
D1 (superior part)	8 (15%)
D2 (descending part)	12 (23%)
D3 (horizontal part)	10 (19%)
D4 (ascending part)	6 (12%)
Multiple segments	12 (23%)
Cannot be determined	4 (8%)
*Surgery with curative intent*,*n* (%)	32 (62%)
*Resection*	26
*No resection*	6
Local invasion	3
Metastases	2
Local invasion + Metastases	1
*Primary palliative treatment*,*n* (%)	20 (38%)

### CECT parameters

Scan protocols were variable because the study covers a long time range and 75% (39/52) of patients were referred with a CECT scan from other non-academic hospitals. Additionally, in clinical practice most CECT scans are performed because of a specific symptoms, not targeted at DA specifically, usually resulting in a CECT with only a PVP. In our dataset in 14 cases CECT was not targeted at staging DA specifically, but CECT was performed because of analyses of symptoms like weight loss. In the other 38 cases it was known that a duodenal tumor was present or there was a high suspicion. Thirteen CECT scans dated before 2015 (2000–2004 *n* = 1; 2005–2009 *n* = 3; 2010–2014 *n* = 9) and 39 scans dated after 2015. Table 2 compares parameters between scans dated before and after 2015. Contrast type, concentration, volume, flowrate, and delay were not available in the majority of cases due to the retrospective nature of the study.

**Table 2:** Comparison of scan parameters between scans of before and after 2015

	Before 2015	After 2015
Patients, *n* (%)	13 (25%)	39 (75%)
* Available contrast phases, n (%)*		
PVP only	9 (69%)	19 (49%)
HAP + PVP	1 (8%)	16 (41%)
PVP + DP	0 (0%)	1 (3%)
HAP + PVP + DP	3 (23%)	3 (8%)
Slice thickness, mm (median, range)	3.0 (1.0–5.0)	2.5 (0.5–5.0)
Ingestion of positive oral contrast,* n* (%)	6 (69%)	12 (31%)
CT of Thorax available,* n* (%)	4 (31%)	26 (67%)

*PVP* portal-venous phase, *HAP* hepatic arterial phase, *DP* delayed phase

### Tumor characteristics on CECT

**Fig. 1:**
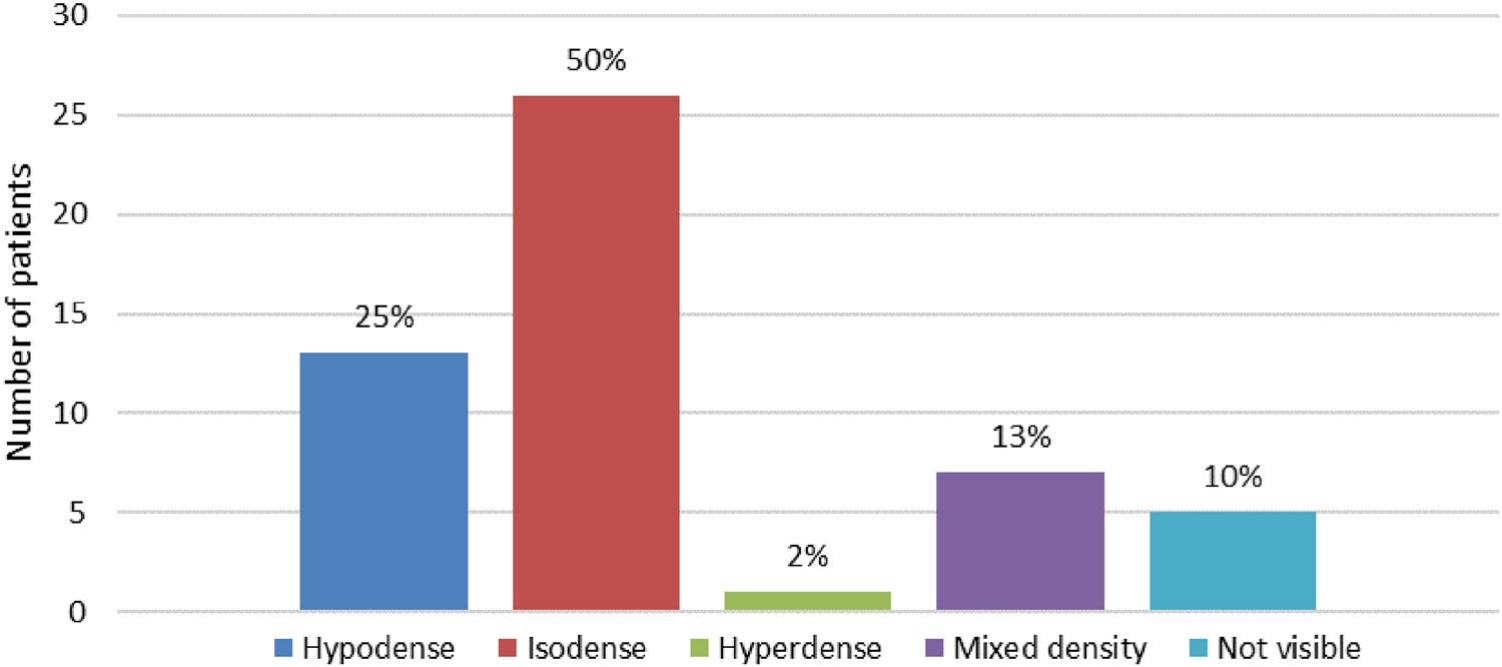
Distribution of tumor enhancement on CECT

**Fig. 2:**
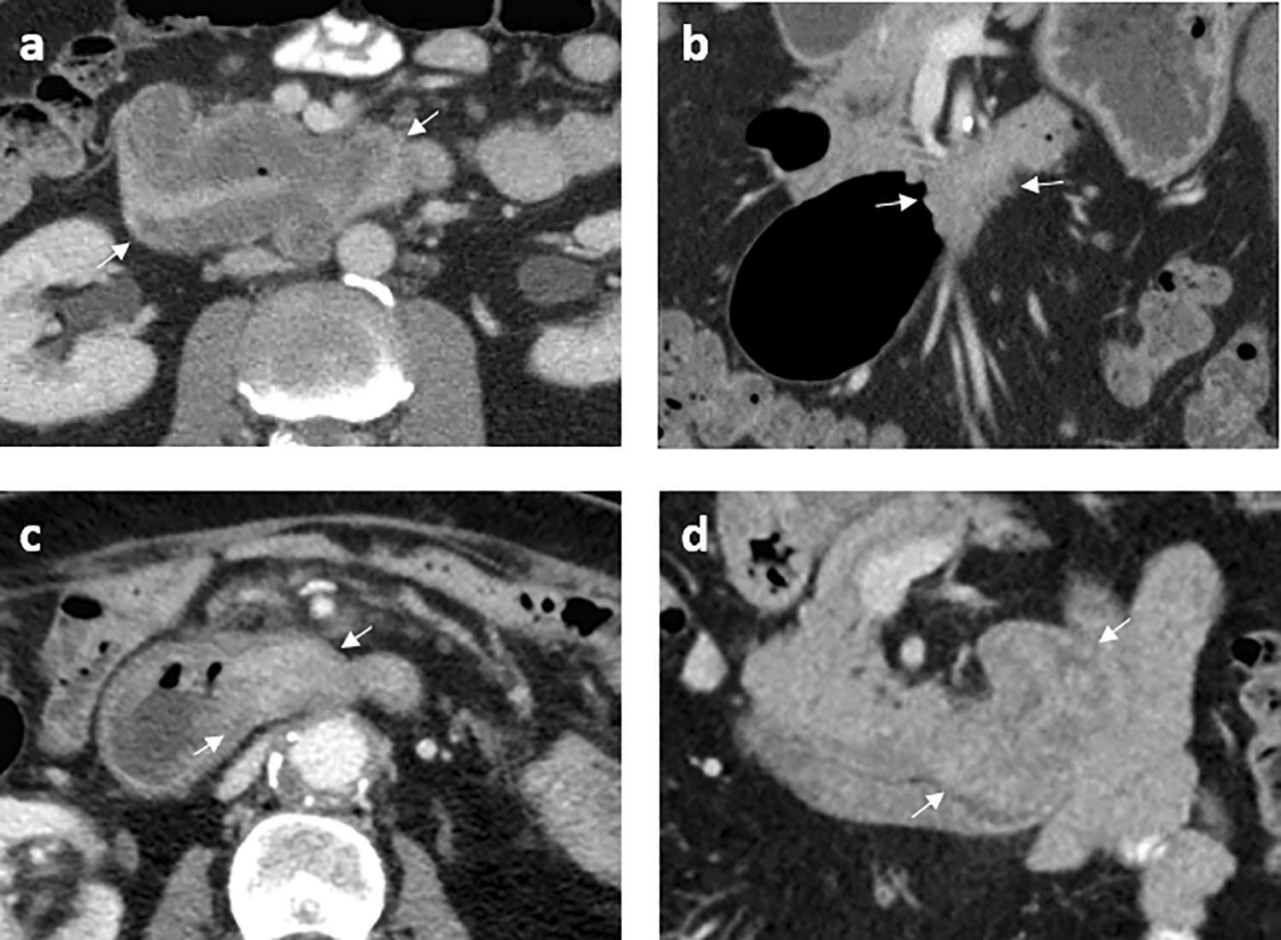
Example for each enhancement pattern on CECT, with the tumor indicated with white arrows. **a** Axial slice with a hypodense tumor. **b** Coronal slice with an isodense tumor. **c** Axial slice with a hyperdense tumor. **d** Coronal slice with a tumor with mixed density

Contrast-enhancement of the tumor compared to normal duodenum on the PVP was classified as; hyperdense, isodense, hypodense, or mixed density. Fifty percent was isodense (26/52) and 10% was not visible on CECT (5/52, Figs. 1 and 2). Tumors mostly involved D2, the descending part (21/52, 40%), with nine of these tumors located in multiple segments including D2.

### Resectability

**Fig. 3:**
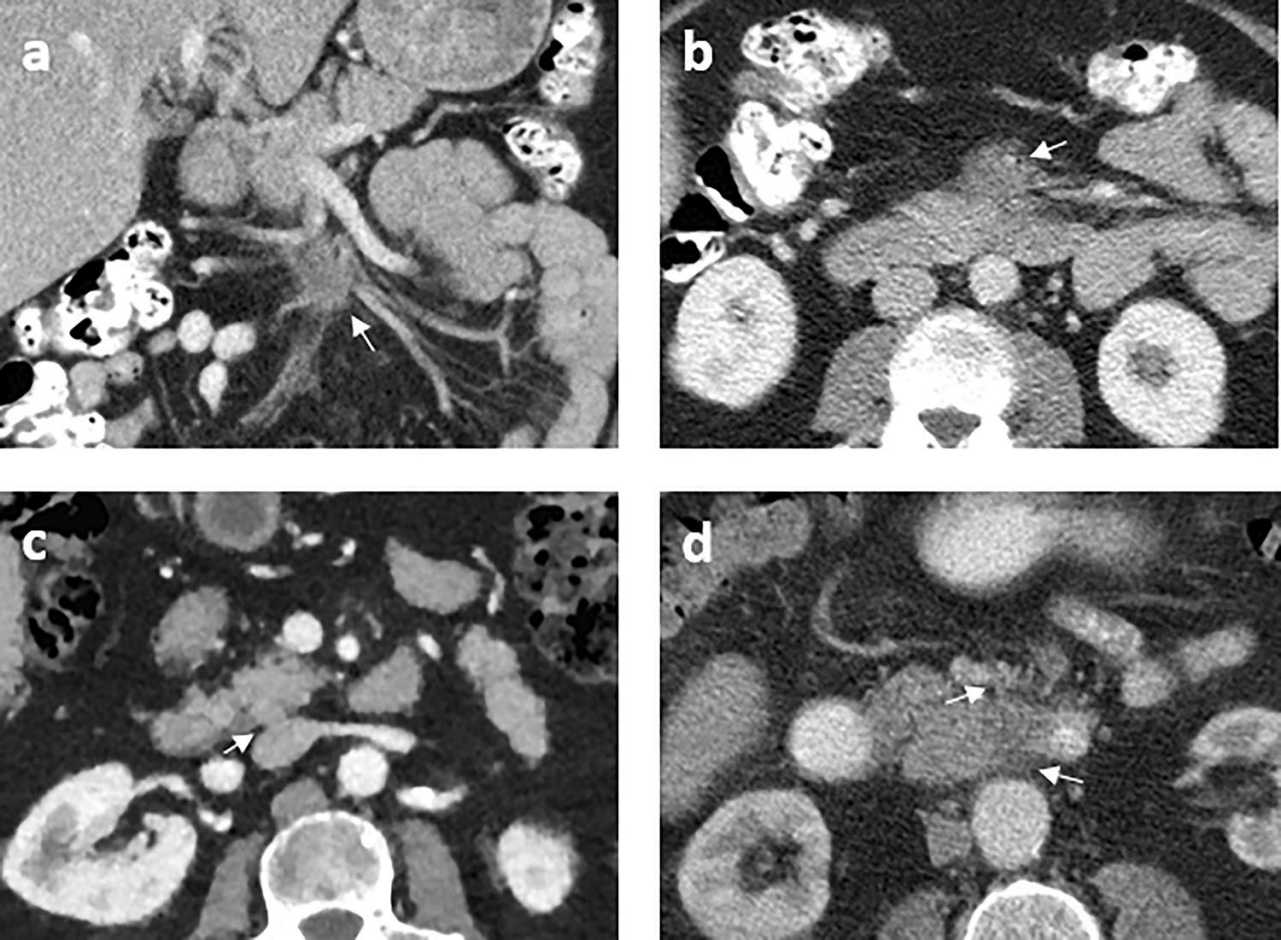
Examples of vascular involvement (indicated with white arrows). **a** Coronal view of a tumor in D3 with involvement of jejunal arteries and veins in the mesenteric root. **b** Axial view of the same tumor as **a**. **c** Axial view of a tumor in D3 with abutment of inferior caval vein. **d** Sagittal view of the same tumor as** c**

Involvement of arteries and/or veins that are included in the DPCG criteria for resectability of pancreatic cancer (SMA, coeliac trunk, CHA, SMV, and portal vein) was seen in 11 patients (11/52, 21%). Vascular involvement of jejunal arteries and/or veins, which are not included in the DPCG criteria but, depending on the extent, can render a patient inoperable, occurred in 15 patients (15/52, 29%). Involvement of the inferior caval vein, also not included in the DPCG criteria, occurred in three patients. Examples are shown in Fig. 3. Seventy-three percent (11/15) of the tumors with vascular involvement of the jejunal arteries and veins involved the third or fourth duodenal segment.

**Fig. 4:**
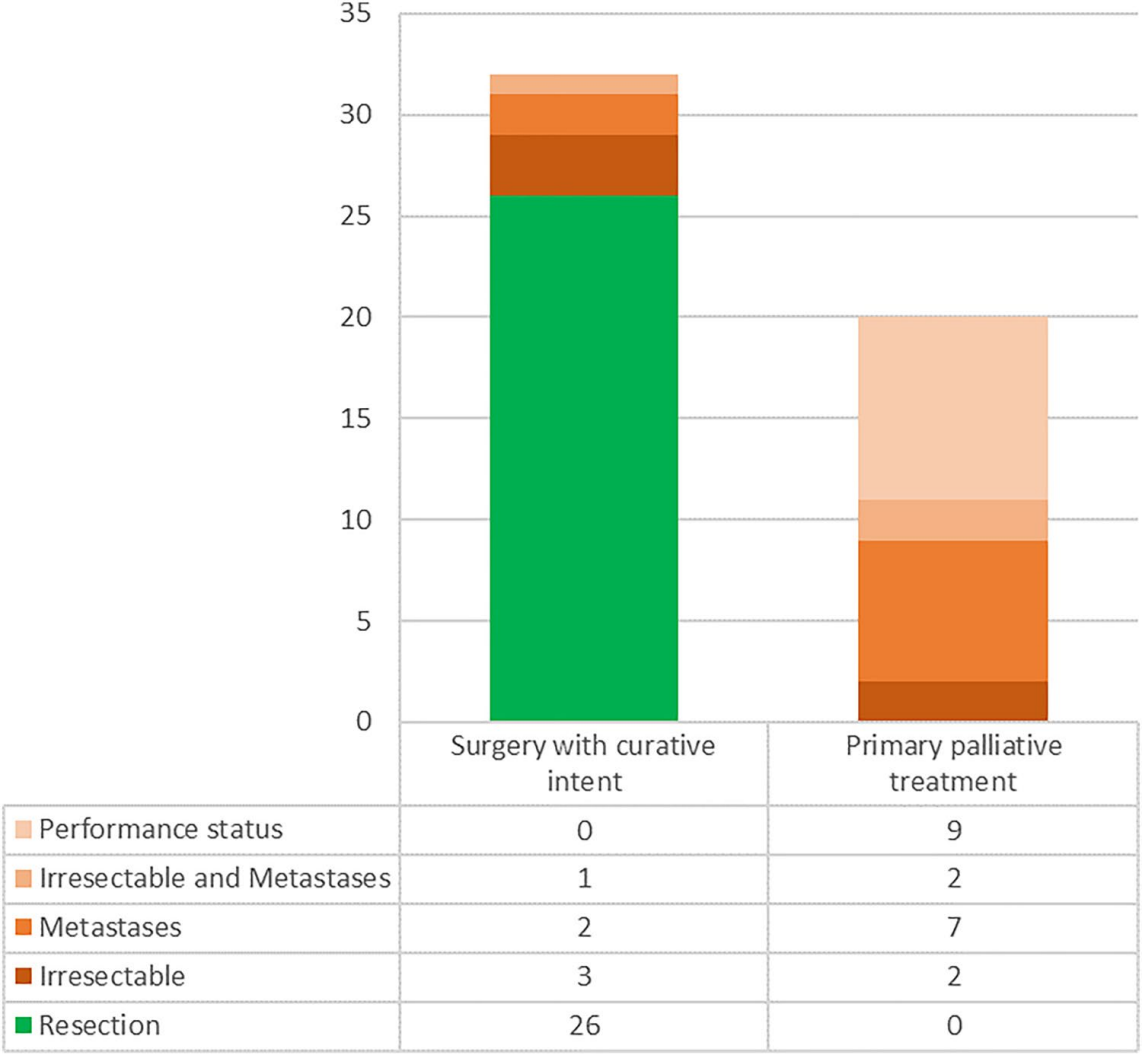
Stacked bar chart representing all patients (*n* = 52). In the left bar, all patients that underwent surgery with a curative intent (*n* = 32) and in the right bar patients with primary palliative treatment (*n* = 20) are displayed. If patients were resected and the reasons patients were not resected are displayed within the bars

Thirty-two patients underwent surgery with a curative intent, of which 26 were resected. Six patients (19%) could not be resected because of unexpected local invasion (4/6) and/or unexpected metastases (3/6). Palliative bypass surgery (gastroenterostomy) was performed in these six patients. On second look in three of these six patients the surgical findings that hampered resection could not be reproduced on preoperative CT scan and in three patients there were in retrospect some indications visible on preoperative CT scan (indeterminate liver lesions or slight vessel induration). Twenty patients received primary palliative treatment, because of metastases (*n* = 7), locally advanced disease (*n* = 2), both (*n* = 2), or insufficient performance status (*n* = 9). Palliative bypass surgery was performed in five of these 20 patients. Data are displayed in Fig. 4.

**Fig. 5:**
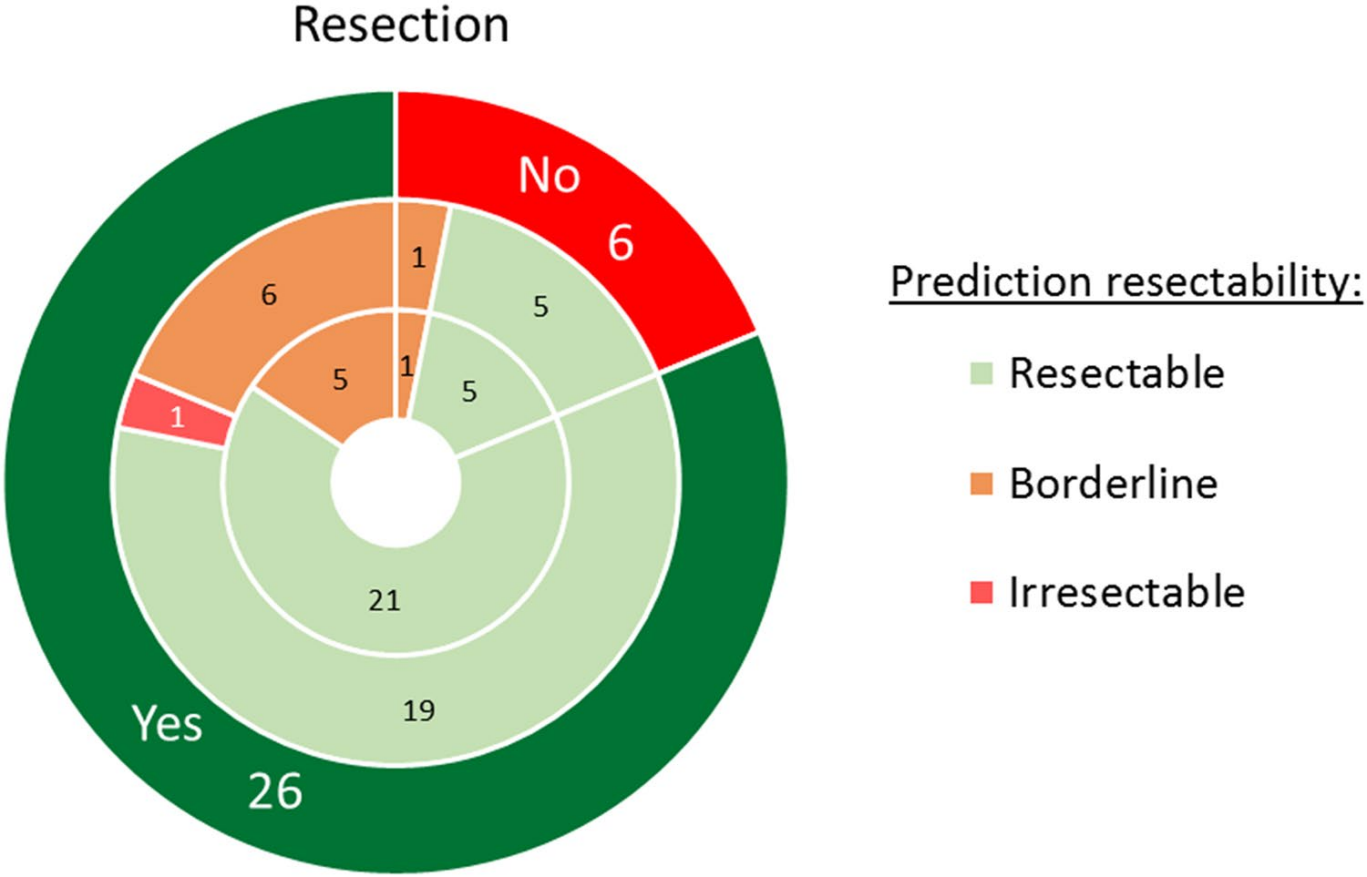
Nested pie chart representing patients that underwent surgery with curative intent (*n* = 32). In the outer circle resection (*n* = 26) vs no resection (*n* = 6). In the middle circle the prediction of the resectability on CECT of the radiologist and in the inner circle of the surgeon

Of the patients that underwent surgery with a curative intent (*n* = 32) the radiologist and surgeon, that individually evaluated resectability, agreed in 88% (*n* = 28) and disagreed in four cases (1 × radiologist irresectable vs surgeon resectable; 2 × radiologist borderline vs surgeon resectable; 1 × radiologist resectable vs surgeon borderline). Most disagreements (*n* = 3) were due to a difference in opinion whether the jejunal vein or artery contact would technically allow a resection or not. In one case there was some induration around the AMS, that the radiologist thought not to be tumor contact but inflammation and the surgeon thought it was tumor contact (at surgery there was involvement of the AMS and of several surrounding organs). Of both experts five patients classified as resectable and one patient as borderline, could unexpectedly not be resected during surgery. One patient classified as irresectable by the radiologist was resected. Data are displayed in Fig. 5.

### TNM-staging on CECT

T- and N-stage on CECT of resected patients (*n* = 26) was correlated to histopathology. The T-stage was correctly classified with CECT in 14 patients (54%), underestimated in seven (27%), overestimated in three (12%), and could not be determined in two patients (8%) (Table 3). In patients with an incorrect T-stage the tumor was more often isodense compared to a correct T-stage (8/10 vs 7/14, p = 0.21).

**Table 3:** Correlation of T-stage on CECT with T-stage at histopathology of resected patients, *n* = 26

CECT	PA		
	T0/1/2	T3	T4
T1/2	*5*^*a*^* (19%)*	5 (19%)	0 (0%)
T3	0 (0%)	*4 (15%)*	2 (8%)
T4	1 (4%)	2 (8%)	*5 (19%)*
Tx	0 (0%)	1 (4%)	1 (4%)

T-stage was correct in 54%, underestimated in 27%, overestimated in 12%, and indeterminate in 8%. a: one patient was classified as T1/2 by CECT after neoadjuvant chemotherapy and as T0 by the pathologist, because there was no residual tumor

Of the resected patients on CECT, there were four patients with N0, eight patients with N1, and 14 patients with N2 and at histopathology there were nine patients with N0, eight patients with N1, and nine patients with N2. The presence (N1 or N2) or absence (N0) of LN metastases was correctly predicted in 11 patients (42%), false positive in two (8%), and false negative in 13 (50%). Sensitivity and specificity for detecting LN metastases on CECT was 24% and 78%, respectively (Table 4).

**Table 4:** Correlation of N-stage using the conventional definition (≥ 10 mm considered malignant) and alternative definition on CECT with N-stage at histopathology of resected patients, *n* = 26

CECT	PA	
	N0	N1/2
N0	*7 (27%)*	13 (50%)
N1/2	2 (8%)	*4 (15%)*

N-stage was correct in 42%, underestimated in 50%, and overestimated in 8%

The results of M-staging for all 52 patients are provided in Table 5. Ten patients were suspected of having metastases: in the liver (*n* = 7), peritoneal (*n* = 2), or at multiple sites (*n* = 1). These were all confirmed by histopathology, ^18^FDG-PET-CT, or follow-up. Additionally, in one patient peritoneal metastases were not visible on CECT. Nine patients showed indeterminate lesions (Mx) on CECT: in the liver (*n* = 7), the lung (*n* = 1), or both (*n* = 1). In one patient, these lesions were confirmed to be metastases; five patients had no metastases according to the reference standard; and in three patients it remained indeterminate. The M-stage was correct on CECT in 42 patients (81%), underestimated in one patient (2%) and unclear in nine patients (17%).

**Table 5:** Correlation of M-stage on CECT to the final M-stage of all patients, *n* = 52, based on histopathology, ^18^FDG-PET-CT, or follow-up

CECT	Final M-stage		
	M0	M1	Mx
M0	*32 (62%)*	1 (2%)	0 (0%)
M1	0 (0%)	*10 (19%)*	0 (0%)
Mx	5 (10%)	1 (2%)	3 (6%)

M-stage was correct in 81%, underestimated in 2%, not overestimated, and unclear in 17%

### Problem solving tools (^18^FDG-PET-CT, MRI, and EUS)

In 23 patients MRI, in 16 patients ^18^FDG-PET-CT, and in five patients EUS was performed. In seven patients (7/23, 30%), MRI provided useful additional information: detection of primary tumor (*n* = 1), confirmation of (suspected) liver metastases (*n* = 3), detection of liver metastases (*n* = 1), and better assessment of tumor extension (*n* = 2). In two patients ,the MRI was false positive for liver metastases. In eight patients (8/16, 50%), ^18^FDG-PET-CT provided useful additional information: detection of primary tumor (*n* = 2), confirmation of (suspected) liver metastases (*n* = 5), and confirmations that suspect liver lesions on CECT were not metastases (*n* = 1). EUS was mainly used to obtain histopathology (*n* = 4) and in one patient the tumor was detected during EUS.

## Discussion

Our study shows that CECT, which is recommended in clinical guidelines, is insufficient for staging and prediction of resectability of duodenal adenocarcinoma. TNM-staging of DA with CECT was correct for T-stage in 54%, N-stage in 42%, and M-stage in 81%. T-stage was frequently underestimated (27%). Sensitivity of CECT for detecting LN metastases was only 24% and specificity was 78%. M-stage with CECT was correct in 81%, but a substantial number of patients (9/52, 17%) had indeterminate liver or lung lesions.

Nine-teen percent (6/32) of patients, who underwent surgery with curative intent, could not be resected due to unexpected local vessel invasion and/or metastases. This is in line with a study on diagnostic laparoscopy in patients with radiologically resectable DA on CECT, where 16% (6/38) was irresectable due to vascular invasion or metastases [23]. In our study the surgeon was more optimistic about resectability and was more frequently correct in resectability prediction than the radiologist. Involvement of jejunal veins and/or arteries was common (15/52), especially in tumors involving the third and fourth duodenal segment. This is in accordance with a study by Stell et al. that also found that lesions of the third and fourth segment duodenum are less often resectable due to invasion of the small bowel mesentery [24]. A lesson learned from our study is that besides vascular contact of the main surrounding arteries and veins that are included in the DPCG criteria, the first jejunal artery and vein are relevant to determine resectability, especially in tumors of the third and fourth segment of the duodenum. In case of near complete encasement of the first jejunal artery and vein, a tumor could be considered as irresectable. However, if after peroperative clamping of the first jejunal artery a limited segment of bowel is affected we consider this as resectable.

In our study, tumor enhancement on CECT was isodense to normal duodenum in 50% of the patients. No tumor could be visualized on CECT in 10%. These findings are comparable with a study analyzing presurgical imaging for resectable periampullary cancer [25]. The inability to visualize the tumor on CECT was not related to size or location. The low performance of CECT to determine T-stage might be explained by the high number of isodense tumors, which were more likely to be staged incorrectly, although this was not statistically significant. Imaging techniques aimed at visualizing the duodenum and tissue characteristics could potentially improve tumor detection, characterization, and staging. For example hypotonic CT with negative or neutral oral contrast agent has shown good results for T-staging of gastric cancer [26, 27]; and hypotonic MRI duodenography with water ingestion resulted in good visualization of the duodenal lumen and wall in healthy volunteers [28]. Hypotonic duodenography could also be used to asses if a tumor is annular or has a polypoid morphology. In a prospective study comparing MRI-enterography to CT-enterography in 150 patients with suspected small bowel disease, MR was more accurate for neoplastic diseases [29]. However, no patients with DA were included in any of these studies. Dual Energy CT is an emerging technique with promising results in other abdominal malignancies. For example the assessment of early versus advanced stage gastric cancer, and the distinction of histological origin of carcinomas in the ampullary region [30, 31].

In our study in some patients ^18^FDG-PET-CT (8/16) and MRI (7/23) provided additional useful information, but MRI also falsely indicated liver metastases in two patients. For the detection of LN metastases, USPIO-enhanced MRI (ultra-small superparamagnetic iron oxide) has proven to be valuable in solid tumors, like prostate and breast cancer [32, 33], but no data are available for small bowel cancers. Diffusion-weighted MR imaging (DWI) has shown to be useful for detecting distant metastases in colorectal cancer [34], but again no data for DA are available. While ^18^FDG-PET-CT may be useful for diagnosing and staging of DA, only a small subset of patients in our study underwent ^18^FDG-PET-CT and literature about ^18^FDG-PET-CT for DA is limited to case series and case reports [35, 36]. ^68^ Ga-FAPI-PET-CT is a new technique that has shown promising results in the detection of the primary tumor, lymph node metastases, and distant metastases in gastrointestinal cancers, with a higher sensitivity compared to conventional ^18^FDG-PET-CT [37].

Duodenal adenocarcinoma is a rare tumor, but literature shows that DA is different in behavior and outcome compared to periampullary or small bowel adenocarcinomas [6, 38–40]. Studies focusing on diagnostics of DA are rare and the number of patients with DA included in diagnostic studies is low. To our knowledge, this study is the only study within the past decades that systematically analyzed the performance of CECT for staging of DA specifically. In 1992 Kazerooni published a study about CT for duodenal neoplasms in 25 patients. However, only eight patients with DA were included and the study mainly focused on differentiating benign from malignant neoplasms [41]. Furthermore, we included patients with all stages of DA, in contrary to many other studies where only resected patients, which are the minority, are included. Including all stages allows for better generalization of results for all DA patients. A limitation is the retrospective nature of the study and a long time range of included patients, which led to a variation of CECT protocols. However, 75% of the CECT scans dated after 2015, the scan year and the availability of an HAP did not seem to influence the performance for staging, but the groups were small (data not shown). Furthermore, variation in scanning protocols is a reflection of daily clinical practice.

## Conclusion

Our study shows that routine CECT is limited for staging and determining resectability in patients with duodenal adenocarcinoma. In 19% of patients, the tumor could not be resected due to unexpected local vessel invasion and/or metastases. Radiologists and clinicians have to be aware that staging of DA with CECT is inadequate, for that reason additional imaging techniques are necessary. This could include CT or MRI with hypotonic duodenography, DWI-MR, USPIO-enhanced MRI, ^18^FDG-PET-CT, or ^68^ Ga-FAPI-PET-CT. Based on the knowledge and experience obtained with this study, we would advise a multiphase hypotonic duodenography CT combined with an MRI scan as the most optimal workup for DA. We implemented this strategy in our hospital for future patients with (suspected) DA. However, this strategy has not been adequately investigated and therefore a multicenter trial is needed to prove the validity of this imaging strategy.

## Abbreviations

CECT: Contrast-enhanced computed tomography
CHA: Common hepatic artery
DA: Duodenal adenocarcinoma
DP: Delayed phase
DPCG: Dutch Pancreatic Cancer Group
DWI: Diffusion-weighted imaging
EUS: Endoscopic ultrasound
^18^FDG-PET-CT: Fluorine-18-fluorodeoxyglucose positron emission tomography computed tomography
^68^ Ga-FAPI-PET-CT: Gallium-68-conjugated FAP inhibitor positron emission tomography computed tomography
HAP: Hepatic arterial phase
LN: Lymph node
MRI: Magnetic resonance imaging
OS: Overall survival
TNM: Tumor node metastases
USPIO: Ultra-small superparamagnetic iron oxide
PVP: Portal-venous phase
SMA: Superior mesenteric artery
SMV: Superior mesenteric vein
